# Circular RNA circ_0048764 promotes the development of breast cancer by regulating microRNA-1296-5p/tripartite motif containing 14 axis

**DOI:** 10.1080/21655979.2021.1995990

**Published:** 2022-01-16

**Authors:** Fei Xie, Yuyuan Xiong, Jiayin Yan, Ling Wang, Wei Yan

**Affiliations:** Department of General Surgery, Xiangyang Central Hospital, Xiangyang, China

**Keywords:** Breast cancer, circ_0048764, miR-1296-5p, Trim14

## Abstract

Breast cancer (BC) is one of the leading causes of cancer-related deaths in females. Circular RNA (circRNA), as reported, is involved in the progression of BC. This work focuses on clarifying the biological function of circ_0048764 in BC and its hidden mechanism. Quantitative real-time polymerase chain reaction (qRT-PCR) was performed to detect the expressions of circ_0048764, microRNA-1296-5p (miR-1296-5p), and tripartite motif containing 14 (TRIM14) in BC tissues and cell lines. Besides, the status of proliferation, migration, invasion and apoptosis of BC cells was probed by cell counting kit-8 (CCK-8), EdU, transwell and flow cytometry assays. Western blot was adopted to examine the level of TRIM14 protein in BC cells. In addition, dual-luciferase reporter gene assay and RNA immunoprecipitation (RIP) assay were conducted to corroborate the targeting relationships between miR-1296-5p and circ_0048764 or TRIM14. It was revealed that circ_0048764 expression was remarkably up-regulated in BC tissues and cells, and circ_0048764 expression was associated with TNM stage and tumor size. Functionally, overexpression of circ_0048764 significantly promoted BC cell proliferative, migrative and invasive abilities and inhibited apoptosis, while circ_0048764 knockdown exerted the opposite effects. Mechanistically, circ_0048764 directly targeted miR-1296-5p and could negatively modulate its expression in BC cells. Besides, miR-1296-5p could reverse the influence of circ_0048764 on BC viability, migration, invasion and apoptosis. Moreover, TRIM14 was confirmed to be a downstream target of miR-1296-5p. Circ_0048764 positively regulated TRIM14 expression in BC cells via targeting miR-1296-5p. Collectively, it is concluded that circ_0048764 promotes the development of BC via modulating the miR-1296-5p/TRIM14 axis.

## Introduction

Breast cancer (BC) is a common malignancy in female globally [1]. Reportedly, the overall 5-year survival rate of BC patients with distant metastasis is only about 25% [2,3]. Despite a series of treatment such as surgery, endocrine therapy, chemotherapy, radiotherapy and targeted therapy for BC in recent years, the recurrence rate and mortality of BC remain high [4]. Thus, it is vital to further delve into the pathogenesis of BC and look for effective new targets for the diagnosis and treatment of BC.

Circular RNAs (circRNAs), the RNA transcripts with covalently closed-loop structures, are deprived of 5 ‘end caps and 3ʹ end tails; specifically, circRNAs are more stable than linear RNAs due to their circular structure, and they are widespread in eukaryotes [5]. In addition, circRNAs have shown significant superiority as biomarkers for various diseases, and accumulating evidence suggests that circRNAs are crucial modulators in the progression of multiple malignancies including BC [6–9]. For example, circ_0025202 expression is markedly reduced in BC tissues and cells, and circ_0025202 overexpression restrains BC cell growth, colony formation, and migration, while promoting apoptosis and increasing the sensitivity to tamoxifen [8]. High expression of circ_001783 is interrelated with the unfavorable prognosis of BC patients, and overexpression of circ_001783 strengthens the proliferative and invasive capabilities of BC cells [9].

MicroRNAs (miRNAs), highly conserved small non-coding RNA transcripts with 18–25*nt* in length, can modulate target genes’ expression via promoting mRNA degradation or restraining translation [10]. MiRNAs can partake in regulating the progression of a variety of cancers, including BC [11–13]. Importantly, circRNAs can function as competitive endogenous RNAs (ceRNAs) to bind and adsorb miRNA specifically [14]. For example, circ_0000388 works as a ceRNA of miR-337-3p to modulate transcription factor 12 (TCF12) expression and promote the progression of cervical cancer [15]. Circ_0026123 depletion represses the multiplication and metastasis of ovarian cancer cells via the miR-124-3p/enhancer of zeste 2 polycomb repressive complex 2 subunit (EZH2) pathway [16]. In addition, in BC, some miRNAs including miR-487a, miR-1271, and miR-548p have been reported to be regulated by circRNAs [17–19]. It has been reported that miR-1296-5p is under-expressed in BC tissues and overexpression of miR-1296-5p inhibits BC cell proliferation [20]. Instead, the mechanism of miR-1296-5p dysfunction in BC has not been completely clarified.

In this work, it was revealed that circ_0048764 was up-regulated in BC tissues by analyzing the Gene Expression Omnibus (GEO) dataset GSE165884. Based on the results of bioinformatics analysis, we hypothesized that circ_0048764 was an oncogenic circRNA in BC progression; besides, circ_0048764 could modulate tripartite motif containing 14 (TRIM14) expression through sponging miR-1296-5p. This study was performed to validate the hypothesis mentioned above. For the first time, in the present study, circ_0048764 was identified as an oncogenic factor, and its downstream mechanism was partly explained. Our study deepens the understanding on BC pathogenesis.

## Materials and methods

### Ethics statement and clinical tissue samples

BC tissues and adjacent normal tissues were provided by 52 patients who underwent mastectomy surgery at Xiangyang Central Hospital. All specimens were pathologically diagnosed as BC, and all subjects did not receive radiotherapy or chemotherapy before the surgery. BC tumor tissue and adjacent normal tissue were frozen at −196°C in liquid nitrogen after surgical resection. The Ethics Committee of Xiangyang Central Hospital approved this work (approval no. XYZX20200605) with the written informed consent from each of the patients.

### Cell culture and transfection

Human embryonic kidney cells (HEK293T), human BC cell lines (MCF7, SKBR3, MDA-MB-231) and human mammary epithelial cell line MCF-10A were available from the American Type Culture Collection (Rockville, MD, USA). These cells were cultured in Dulbecco’s modified Eagle’s medium (Beyotime, Shanghai, China) with 10% fetal bovine serum (FBS; Beyotime, Shanghai, China), 100 U/mL penicillin and 100 μg/mL streptomycin (Beyotime, Shanghai, China) at 37°C in 5% CO_2_. Circ_0048764 overexpression plasmid (pcDNA-circ_0048764), empty vector cDNA (pcDNA-NC), small interfering RNAs (siRNAs) targeting circ_0048764 (si-circ_0048764-1 and si-circ_0048764-2), siRNA negative control (scramble siRNA, si-NC), miR-1296-5p mimics and its control (mim-NC), miR-1296-5p inhibitors and its control (Inh-NC) were produced by GenePharma (Shanghai, China). The above vectors were subsequently transfected into SKBR3 and MCF7 cells by Lipofectamine®3000 (Invitrogen, Carlsbad, CA, USA). 24 h later, quantitative real-time polymerase chain reaction (qRT-PCR) was executed to detect the efficacy.

### qRT-PCR

Total RNA from tissues and cells was extracted by TRIzol reagent (Invitrogen, Shanghai, China), with RNA concentration and purity probed by NanoDrop2000 (Thermo Scientific, Waltham, MA, USA). Besides, total RNA was reversely transcribed by a First Strand cDNA Synthesis Kit (Thermo Fisher Scientific Inc., Rockford, IL, USA). In addition, qRT-PCR was performed on an ABI7300 system (Thermo Fisher Scientific Inc.) with a SYBR® Premix-Ex-Taq ™ kit (Takara, Tokyo, Japan). mRNA expression and cricRNA expression were normalized by GAPDH, and miRNA expression was normalized by U6. Subcellular localization analysis of circ_0048764 was performed with a PARIS™ kit (Thermo Fisher Scientific, Waltham, MA, USA). After the RNA in the cytoplasm and nucleus of SKBR3 and MCF7 cells was respectively extracted, the expression of circ_0048764 in the cytoplasm and nucleus of SKBR3 and MCF7 cells was detected by qRT-PCR, with GAPDH and U6 as controls for cytoplasm and nucleus, respectively.

The specific primer sequences:

Circ_0048764 Forward: 5ʹ-AAGAAAGACTGAGCCCCTCCCCTGCCC-3ʹ,

Circ_0048764 Reverse: 5ʹ-GTCCTCTGTGCAGGTTGGAGGATGGTT-3ʹ;

miR-1296 Forward 5p: 5ʹ-GTTAGGGCCCTGGCTCC-3ʹ,

miR-1296 Reverse 5p: 5ʹ-CAGTGCGTGTCGTGGAGT-3ʹ;

TRIM14 Forward: 5ʹ-GCAGAGACAGAGCTAGACTGTAAAGGT-3ʹ,

TRIM14 Reverse: 5ʹ-CCTGGTCACAATTGATATGGA-3ʹ;

U6 Forward: 5ʹ-CTCGCTTCGGCAGCACA-3ʹ,

U6 Reverse: 5ʹ-AACGCTTCACGAATTTGCGT-3ʹ;

GAPDH Forward: 5ʹ-GCACCGTCAAGGCTGAGAAC-3ʹ,

GAPDH Reverse: 5ʹ-TGGTGAAGACGCCAGTGGA-3ʹ.

### Cell Counting Kit-8 (CCK-8) assay

SKBR3 or MCF7 cells were inoculated into 96-well plates with 100 μL of cell suspension per well (2 × 10^4^ cells/mL). Then the cells were routinely cultured. 24 h later, 10 μL of CCK-8 solution (MedChemExpress, Monmouth Junction, NJ, USA) was loaded into each well, and the cells were incubated for 1 h at 37°C. Ultimately, the value of optical density (OD) at 450 nm wavelength was determined by a microplate reader. With the same method, the OD values of the cells were measured at

### EdU assay

Cell viability was detected by an EdU kit (Beyotime, Shanghai, China) according to the manufacturer’s instruction. Transfected SKBR3 and MCF7 cells were inoculated in 24-well plates and cultivated for 24 h at 37°C. After that, 200 μL of 50 μM EdU solution was loaded and the cells incubated for 2 h at 37°C. Next, the medium was discarded, and the cells were fixed with paraformaldehyde and then incubated with 200 μL of 2 mg/mL glycine for 5 min. Next, the cells were permeabilized with 100 μL of 0.5% Triton X-100 in phosphate buffer saline (PBS). After the cell were rinsed with phosphate buffer saline (PBS) twice for 2 min each time, the cells were stained with Apollo staining solution at ambient temperature for 30 min in darkness. Subsequently, the cells were incubated with DAPI staining solution for 20 min to stain the nuclei. Finally, the cells were photographed and subsequently counted under a fluorescent microscope.

### Transwell assay

Transwell assay was subsequently conducted to probe cell migrative and invasive abilities with Transwell inserts (Costar, Cambridge, MA, USA). 5 × 10^4^ transfected BC cells were suspended in 200 μL of serum-free medium and transferred into the upper chamber. Meanwhile, the lower chamber was filled with 500 μL of medium containing 10% FBS. Then the cells were cultured. 24 h later, the cells in the upper chamber were gently wiped off with a cotton swab. Then, the cells were fixed with formaldehyde, subsequently stained with crystal violet solution for 15 min and rinsed twice with PBS. After air-drying, five fields were randomly photographed under an inverted microscope (200 ×), and subsequently, cells counting was performed. The membrane of upper chambers was pre-coated with a layer of Matrigel (1:10; BD Biosciences, Franklin Lakes, NJ, USA), which was used in the invasion experiment, but not for migration assay.

### Flow cytometry for apoptosis

The apoptosis of BC cells was evaluated by a FITC-Annexin V/propidiumiodide (PI) double staining kit (Invitrogen, Carlsbad, CA, USA). SKBR3 or MCF7 cells at logarithmic growth phase were trypsinized and washed with PBS. Subsequently, for each sample, 1 × 10^5^ cells were resuspended in 200 μl of Annexin V-binding buffer and then stained with 5 μL of Annexin V-FITC staining solution and 5 μL of propidium iodide (PI) staining solution for 15 min at ambient temperature in darkness. The cell suspension was diluted in Annexin V-binding buffer, and subsequently the stained cells were detected by a flow cytometer (BD Biosciences, Bedford, MA, USA). Data analysis was performed by FlowJo software (TreeStar, San Carlo, CA, USA).

### RNA immunoprecipitation (RIP)

RIP assay was accomplished by the Magna RIP RNA-binding protein immunoprecipitation kit (Millipore, Billerica, MA, USA) according to the manufacturer’s instruction. SKBR3 and MCF7 cells were lysed in RIP lysis buffer, and the cell extract was specifically incubated with RIPA buffer, which contained magnetic beads conjugated with human anti-Argonaute 2 (Ago2) antibody or immunoglobulin G (IgG) antibody at 4°C for 12 h. Next, the complex containing the magnetic beads was rinsed with RIP buffer and subsequently incubated with proteinase K for 30 min at 55°C. Ultimately, immunoprecipitated RNA was extracted, and qRT-PCR was performed to detect the enrichment of circ_0048764 and miR-1296-5p.

### Dual- luciferase reporter gene assay

Wild type binding sequences or mutated sequences between miR-1296-5p and circ_0048764 or miR-1296-5p and TRIM14 mRNA 3′UTR predicted by bioinformatics were respectively amplified and subsequently cloned into pmirGLO Dual-Luciferase miRNA Target Expression Vector (Promega, Madison, WI, USA) to generate wild-type (circ_0048764 WT and TRIM14 WT) or mutant type (circ_0048764 MUT and TRIM14 MUT) luciferase reporter gene vectors. Next, the above vectors and miR-1296-5p mimics, miR-1296-5p inhibitors or their negative controls were co-transfected into HEK293T cells, respectively. 48 h later, the relative activity was estimated by the dual luciferase reporter gene assay system (Promega, Madison, WI, USA).

### Western blot

BC cells were lysed with radioimmunoprecipitation assay (RIPA) lysate (Beyotime, Shanghai, China) and the supernatant was immediately obtained by high-speed centrifugation to extract total protein, with the total protein concentration examined by a BCA protein assay kit (Beyotime, Shanghai, China). The protein was subsequently heated at 100°C for 10 min. Specifically, proteins were separated by sodium dodecyl sulfate polyacrylamide gel electrophoresis (SDS-PAGE) and transferred to polyvinylidene difluoride (PVDF) membranes (Millipore, Bedford, MA, USA), which were subsequently blocked with 5% skimmed milk for 1 h and rinsed three times with Tris Buffered Saline with Tween 20 (TBST). Proteins were subsequently incubated with primary anti-TRIM14 antibody (PA5-50806, 1:1000, Thermo Fisher Scientific) and anti-GAPDH antibody (ab181602, 1:1500, Abcam, Shanghai, China) overnight at 4°C. After the membrane was rinsed by TBST again, the PVDF membrane was incubated with Goat Anti-Rabbit IgG H&L (ab150077, 1:5000, Abcam, Shanghai, China) for 1 h at room temperature. Ultimately, proteins were specifically visualized by a Novex™ ECL Chemiluminescent Substrate Reagent Kit (Thermo Fisher Scientific), with GAPDH as the internal reference.

### Statistical analysis

The statistical analysis was conducted by SPSS 23.0 statistical analysis software (SPSS Inc., Chicago, IL, USA). Quantitative data were expressed as ‘mean ± standard deviation’. Kolmogorov-Smirnov test was used to examine the normality and equal variance of the data. Besides, *t*-test was employed for comparison between the two groups, and one-way analysis of variance and Tukey’s post-hoc test were used for comparisons among multiple groups. If the data were skewed distributed, the comparisons were made by Wilcoxon signed-rank test. The association between circ_0048764 expression and clinicopathological parameters of BC were analyzed by the chi-square test. Ultimately, the correlation between groups was evaluated by Pearson’s correlation coefficient. Statistically, *P* < 0.05 is meaningful.

## Results

The present work was aimed to clarify the expression characteristics, biological function, and downstream mechanism of circ_0048476 in BC. A series of bioinformatics analysis, gain-of-function experiments, loss-of-function experiments and mechanism investigation were performed.

### Circ_0048764 is in high expression in BC tissues

**Figure 1.**
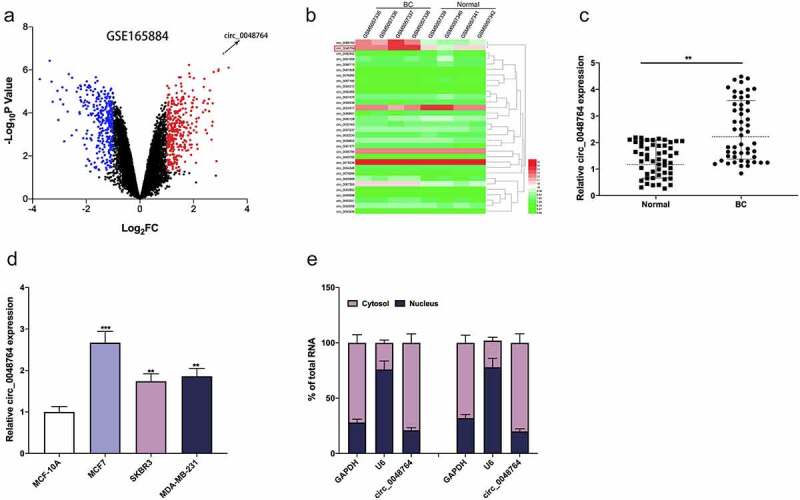
Circ_0048764 is up-regulated in BC tissues and cells (a) the GSE165884 dataset was analyzed by the GEO2R online tool to show the expression changes of circRNAs in BC tissues and normal tissues, with red representing the upregulated circRNAs and blue representing the downregulated circRNAs. (b) A heat map showed the differential expression of circRNAs in BC tissues and normal tissues. (c and d) qRT-PCR was used to detect the expression of circ_0048764 in BC tissues (c) and cell lines (d). Nucleocytoplasmic separation assay was used to detect the subcellular localization of circ_0048764 in SKBR3 and MCF7 cells. **P < 0.01, and ***P < 0.001.

GSE165884 dataset was downloaded from GEO database, and the differentially expressed circRNAs were analyzed by GEO2R. The cutoff criteria were set: log_2_ fold change > 1, and *P* < 0.05. It was revealed that circ_0048764 expression in BC tissues was markedly up-regulated as against that in normal tissues (Figure 1(a,b)). Subsequently, circ_0048764 expression in tumor tissues and normal tissues of BC patients was further detected by qRT-PCR, the results of which suggested that circ_0048764 was highly expressed in BC tissues as against that in normal tissues (Figure 1(c)). Notably, chi-square test highlighted that high expression of circ_0048764 was associated with higher TNM stage and larger tumor size of BC patients (Table 1). Additionally, circ_0048764 expression was remarkably raised in BC cell lines (MCF7, SKBR3, and MDA-MB-231) as against that in normal mammary epithelial cell line MCF-10A (Figure 1(d)). Furthermore, the subcellular distribution of circ_0048764 in BC cells was analyzed, and it was found that circ_0048764 was predominantly distributed in the cytoplasm of SKBR3 and MCF7 cells (Figure 1(e)), suggesting that it could probably function as a ceRNA.

**Table 1.** The correlation between clinicopathological features and expression of circ_0048764 in BC patients

Pathological Parameters	Numbers (n = 52)	Circ_0048764 expression		*P*-Value
		High (n = 26)	Low (n = 26)	
Age (years)				0.4022
< 45	23	10	13	
≥ 45	29	16	13	
Menopause				0.2542
Yes	20	12	8	
No	32	14	18	
ER status				0.7813
Negative	27	13	14	
Positive	25	13	12	
PR stage				0.5745
Negative	22	10	12	
Positive	30	16	14	
HER2 stage				0.2658
Negative	28	12	16	
Positive	24	14	10	
TNM stage				0.0110*
I + II	21	6	15	
III + IV	31	20	11	
Tumor size (cm)				0.0124*
< 5	27	9	18	
≥ 5	25	17	8	

* *P* < 0.05

### Circ_0048764 promotes the proliferative, migrative, and invasive abilities, and inhibits the apoptosis of BC cell

**Figure 2.**
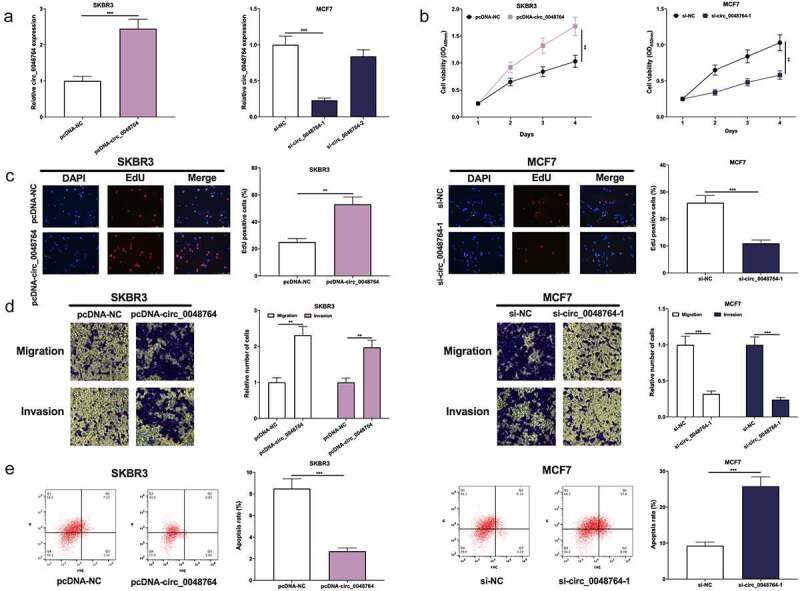
Circ0048764 promotes BC cells’ proliferation, migration and invasion and inhibits apoptosis (a) pcDNA-NC or pcDNA-circ_0048764 was transfected into SKBR3 cells and si-NC, si-circ0048764-1 and circ_0048764-2 were transfected into MCF7 cells, and the expression of circ_0048764 in SKBR3 and MCF7 cells after transfection was detected by qRT-PCR. (b and c) CCK-8 and EdU assays were used to detect the effects of overexpression or knockdown of circ_0048764 on the proliferation of SKBR3 and MCF7 cells. (d) transwell assay was used to detect the effects of overexpression or knockdown of circ_0048764 on SKBR3 and MCF7 cells’ migration and invasion. (e) flow cytometry was used to detect the effect of overexpression or knockdown of circ_0048764 on the apoptosis of SKBR3 and MCF7 cells. ** *P* < 0.01, and ***P < 0.001.

Circ_0048764 expression was the lowest in SKBR3 cells and highest in MCF7 cells among BC cell lines. Therefore, circ_0048764 overexpression plasmid (pcDNA-circ_0048764) was transfected into SKBR3 cells, and si-circ_0048764-1 and si-circ_0048764-2 were transfected into MCF7 cells to construct *in-vitro* models of circ_0048764 overexpression and knockdown, respectively (Figure 2(b)). si-circ_0048764-1 was used for subsequent experiments due to its better knockdown efficiency. CCK-8, EdU, Transwell assays and flow cytometry showed that circ_0048764 overexpression significantly accelerated SKBR3 cell growth, migration and invasion and inhibited apoptosis (Figure 2(c-e)); while circ_0048764 knockdown significantly inhibited MCF7 cells’ proliferative, migrative and invasive abilities and induced apoptosis (Figure 2(c-e)).

### Circ_0048764 competitively binds to miR-1296-5p

**Figure 3.**
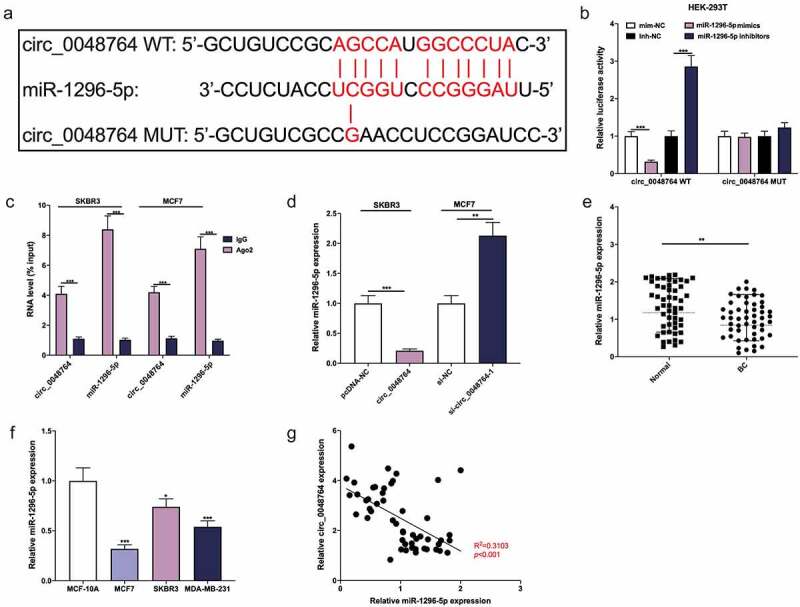
Circ_0048764 adsorbs miR-1296-5p (a) The potential binding site between circ_0048764 and miR-1296-5p was predicted by the Circinteractome database. (b) The interaction between circ_0048764 and miR-1296-5p was detected with a dual-luciferase reporter gene assay with HEK-293 T cells . (c) RIP assay was performed to detect the enrichment of circ_0048764 and miR-1296-5p in the immunoprecipitate of Ago2 group or IgG group. (d) qRT-PCR was used to detect the expression of miR-1296-5p in SKBR3 and MCF7 cells with circ_0048764 overexpression or knockdown. e and f. The expression of miR-1296-5p in BC tissues (e) and cell lines (f) was detected by qRT-PCR. (g) Pearson’s correlation analysis was performed to detect the correlation between circ_0048764 expression and miR-1296-5p expression in BC tissues. * *P* < 0.05, ** *P* < 0.01, and *** *P* < 0.001.

To clarify the downstream mechanism of circ_0048764 in BC, Circinteractome database was searched. It was revealed a binding site between circ_0048764 and miR-1296-5p (Figure 3(a)). Dual-luciferase reporter gene assay indicated that miR-1296-5p up-regulation greatly depressed the luciferase activity of circ_0048764 WT reporter, and miR-1296-5p inhibition significantly worked oppositely, but neither miR-1296-5p up-regulation nor inhibition had an obvious effect on that of circ_0048764 MUT reporter (Figure 3(b)). What’s more, RIP assay suggested that both circ_0048764 and miR-1296-5p were remarkably enriched in the immunoprecipitate of Ago2 group as against that of the IgG group (Figure 3(c)). Additionally, it was revealed that circ_0048764 up-regulation demonstrably restrained miR-1296-5p expression, and circ_0048764 depletion worked oppositely (Figure 3(d)). qRT-PCR highlighted that miR-1296-5p was remarkably inhibited in BC tissues as against normal tissues (Figure 3(e)). MiR-1296-5p was greatly inhibited in BC cell lines as against normal MCF-10A cells (Figure 3(f)). Pearson’s correlation analysis suggested that circ_0048764 was negatively interrelated with miR-1296-5p expression in BC tissues (Figure 3(g)). These data suggested that miR-1269-5p was absorbed by circ_0048764 in BC.

### Circ_0048764 plays a cancer-promoting role in BC by targeting miR-1296-5p

**Figure 4.**
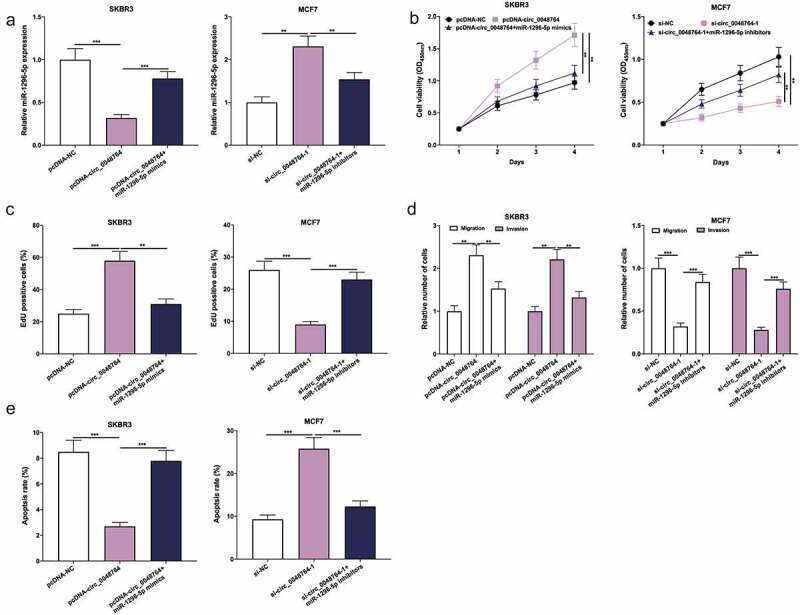
Circ_0048764 promotes BC cell proliferation, migration and invasion, and inhibits apoptosis by targeting miR-1296-5p (a) pcDNA-NC, pcDNA-circ_0048764 and pcDNA-circ_0048764 + miR-1296-5p mimics were transfected into SKBR3 cells, respectively; si-NC, si-circ_0048764-1 and si-circ_0048764-1 + miR-1296-5p inhibitors were transfected into MCF7 cells, respectively, and the expression of miR-1296-5p was detected by qRT-PCR. (b and c) CCK-8 and EdU assays were used to detect SKBR3 or MCF7 cells’ proliferation after transfection. (d) transwell assay was used to detect SKBR3 or MCF7 cells’ migration and invasion after transfection. (e) flow cytometry was used to detect the apoptosis of SKBR3 or MCF7 cells after transfection. ** *P* < 0.01, and ***P < 0.001.

Next, pcDNA-NC, pcDNA-circ_0048764, pcDNA-circ_0048764 + miR-1296-5p mimics were transfected into SKBR3 cells, and si-NC, si-circ_0048764-1, si-circ_0048764-1 + miR-1296-5p inhibitors were transfected into MCF7 cells, respectively (Figure 4(a)). Besides, CCK-8, EdU, transwell assays and flow cytometry highlighted that transfection of miR-1296-5p mimics weakened the promoting effect of overexpression of circ_0048764 on the malignant biological behaviors of SKBR3 cells; knockdown of circ_0048764 inhibited MCF7 cell proliferative, migrative and invasive abilities, and induced apoptosis, but these effects were counteracted by transfection of miR-1296-5p inhibitors (Figure 4(b-f)). These results indicate that circ_0048764 strengthens BC cell viability, migration and invasion, and inhibits apoptosis via targeting miR-1296-5p.

### Circ_0048764 is implicated in BC progression via modulating miR-1296-5p/TRIM14 axis

**Figure 5.**
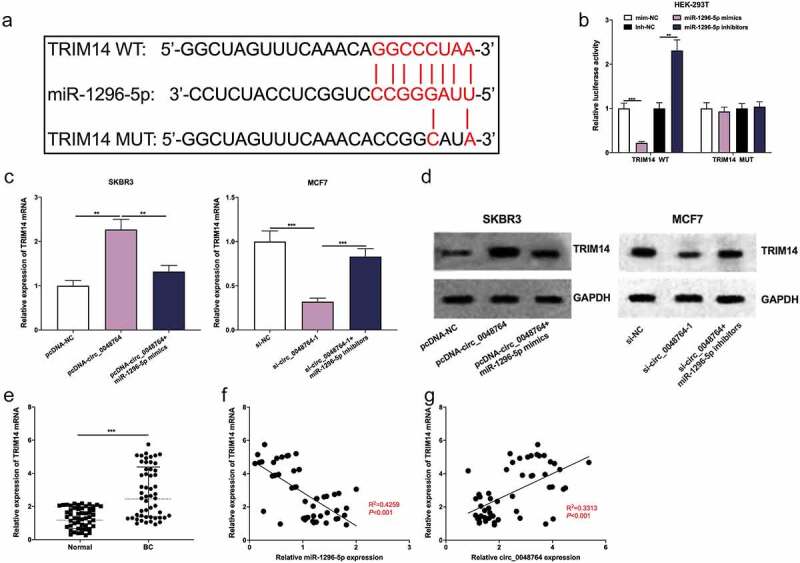
Circ_0048764/miR-1296-5p axis regulates BC progression by regulating the expression of TRIM14 (a)The potential binding site between TRIM14 mRNA 3′ UTR and miR-1296-5p were predicted by targetscan database. (b) dual-luciferase reporter gene assay was used to examine the interaction of TRIM14 mRNA 3’UTR and miR-1296-5p in HEK-293 T cells. (c and d) the levels of TRIM14 mRNA and protein in SKBR3 cells transfected with pcDNA-NC, pcDNA-circ_0048764 and pcDNA-circ_0048764 + miR-1296-5p mimics, or MCF7 cells transfected with si-NC, si-circ_0048764-1 and si-circ_0048764-1 + miR-1296-5p inhibitors were detected by qRT-PCR and western blot assay, respectively. (e) the expression of TRIM14 mRNA in BC tissues and normal tissues was detected by qRT-PCR. (f and g) Pearson’s correlation analysis was used to detect the correlation between TRIM14 mRNA expression and miR-1296-5p expression or circ_0048764 expression in BC tissues, respectively. ** *P* < 0.01, and ***P < 0.001.

Furthermore, the downstream targets of miR-1296-5p were predicted by Targetscan database, and it was found that there was a binding site between TRIM14 mRNA 3ʹUTR and miR-1296-5p (Figure 5(a)). Besides, dual-luciferase reporter gene assay highlighted that up-regulation of miR-1296-5p greatly inhibited the luciferase activity of TRIM14 WT reporter, and inhibition of miR-1296-5p promoted the luciferase activity, but that of TRIM14 MUT was not significantly impacted by the selective regulation of miR-1296-5p (Figure 5(b)). It was also found that up-regulation of circ_0048764 promoted TRIM14 mRNA and protein expressions in SKBR3 cells, while up-regulation of miR-1296-5p weakened this effect (Figure 5(c,d); knockdown of circ_0048764 inhibited TRIM14 mRNA and protein expressions in MCF7 cells, while miR-1296-5p inhibition reversed this effect (Figure 5(c,d)). In addition, qRT-PCR showed that TRIM14 mRNA was highly expressed in BC tissues as against normal tissues (Figure 5(e)). Besides, Pearson’s correlation analysis revealed that TRIM14 mRNA expression was negatively correlated with miR-1296-5p expression and positively correlated with circ_0048764 expression in BC tissues (Figure 5(f,g)).

## Discussion

Reportedly, circRNA is widely expressed in tissues and has a tissue-specific expression pattern. CircRNAs are involved in regulating a variety of biological processes [21,22]. To date, many circRNAs have been identified as biomarkers of cancer, and a growing number of studies have reported that circRNAs feature prominently in carcinogenesis and tumor progression in BC [8,15,23–25]. For example, circFAT1 regulates oxaliplatin resistance of BC cells by modulating miR-525-5p/spindle and kinetochore associated complex subunit 1 (SKA1) axis [24]. Another study reports that overexpression of circRASSF2 promotes breast cancer progression by modulating miR-1205/homeobox A1 (HOXA1) axis, and its high expression indicates poor prognosis [25]. In this work, circRNA microarray analysis implied that circ_0048764 was up-regulated in BC tissues, which was validate by qRT-PCR data based on our own cohort. Meanwhile, circ_0048764 expression was associated with higher TNM stage as well as larger tumor size of patients, suggesting the potential of circ_0048764 as a prognostic biomarker. Additionally, *in-vitro* experiments showed that circ_0048764 overexpression potentiated BC cells’ proliferative, migrative and invasive abilities, but inhibited the apoptosis; knockdown of circ_0048764 exerted the opposite effects. These data support that circ_0048764 may accelerate the progression of BC. To further validate the potential of circ_0048764 as a prognostic biomarker for BC, more patients from different medical centers should be enrolled in the following work, and the relationship between circ_0048764 expression and the survival time of the patients should be evaluated.

MiRNAs have been reported to exert important functions in regulating biological processes such as cell viability, migration and apoptosis [26]. MiRNAs, as reported, are key modulators in BC progression. For instance, miR-141 overexpression suppresses the viability, migration, and invasion of BC cells by restraining acidic nuclear phosphoprotein 32 family member E (ANP32E) expression [27]. Inhibition of miR‑663b can restrain BC cell multiplication and facilitate cell apoptosis, thus enhancing the sensitivity of tamoxifen [28]. CircRNAs, as reported, can act as ceRNAs to modulate the expression of miRNAs. For example, circACAP2 accelerates the growth and metastasis of BC cells via absorbing miR-29a/b-3p and up-regulating collagen type V alpha 1 chain (COL5A1) expression [29]. Circ_0061825 regulates trefoil factor 1 (TFF1) expression and accelerates the progression of BC via adsorbing miR-326 [30]. Herein our data validated that circ_0048764 could competitively bind with miR-1296-5p to repress its expression in BC. Reportedly, miR-1296-5p plays a cancer-suppressive role in gastric cancer, liver cancer, as well as BC, and it can inhibit tumor cell proliferative, migrative, and invasive abilities and induce apoptosis [20,31,32]. In the present study, it was also found that miR-1296-5p expression was inhibited in BC tissues and cells, which is consistent with the findings of the previous study [20]. Importantly, miR-1296-5p could reverse the effect of circ_0048764 on the malignant biological processes of BC cells. These findings highlight that circ_0048764 plays a cancer-promoter in BC by adsorbing miR-1296-5p.

TRIM14 is a member of the TRIM family, which is found to partake in diverse biological processes, such as cell viability, development, apoptosis, and differentiation [33]. Several cancer-related signaling pathways are reportedly activated by TRIM14, such as the AKT pathway in osteosarcoma [34], the NF-κB pathway in tongue squamous cell carcinoma [35], and the Wnt/β-catenin pathway in glioma [36]. Additionally, TRIM14 represses the expression of phosphatase and tensin homology (PTEN) in colorectal cancer [37]. These studies suggest that TRIM14 is a promoting factor for cancer progression. It has been reported that TRIM14 depletion suppresses viability and induces apoptosis of BC cells [33]. In the present study, it was revealed that TRIM14 was a downstream target of miR-1296-5p in BC cells. Besides, circ_0048764 could elevate TRIM14 expression in BC cells by adsorbing miR-1296-5p. These findings suggest that the circ_0048764/miR-1296-5p/TRIM14 axis is a novel ceRNA network, which is implicated in BC progression.

## Conclusion

In summary, circ_0048764 expression level is elevated in BC tissues and cell lines. In addition, circ_0048764 can expedite BC cell growth, migration, and invasion, and inhibit apoptosis via regulating the miR-1296-5p/TRIM14 axis. It is expected to provide new ideas for the diagnosing and treating BC. However, our study was limited to *in-vitro* experiments, and *in-vivo* assays are necessary to further confirm our conclusion. Additionally, other downstream miRNAs of circ_0048764 remain to be clarified in the following studies.

## Data Availability

The data used to support the findings of this study are available from the corresponding author upon request.

## Highlights

- The expression of circ_0048764 is up-regulated in breast cancer;
- Circ_0048764 promotes the malignancy of breast cancer;
- Circ_0048764, miR-1296-5p and TRIM14 forms a ceRNA network to regulate the progression of breast cancer.
